# Nanotechnology in Immunotherapy for Type 1 Diabetes: Promising Innovations and Future Advances

**DOI:** 10.3390/pharmaceutics14030644

**Published:** 2022-03-15

**Authors:** Saumya Nigam, Jack Owen Bishop, Hanaan Hayat, Tahnia Quadri, Hasaan Hayat, Ping Wang

**Affiliations:** 1 Precision Health Program, Michigan State University, East Lansing, MI 48824, USA; nigamsau@msu.edu (S.N.); bishop91@msu.edu (J.O.B.); hayathan@msu.edu (H.H.); quadrita@msu.edu (T.Q.); hayathas@msu.edu (H.H.); 2 Department of Radiology, College of Human Medicine, Michigan State University, East Lansing, MI 48824, USA; 3 Lyman Briggs College, Michigan State University, East Lansing, MI 48824, USA

**Keywords:** autoimmunity, B cells, beta cells, cell therapy, immune checkpoint molecules, immunotherapy, microRNA, nanoparticles, stem cells, T cells, type 1 diabetes

## Abstract

Diabetes is a chronic condition which affects the glucose metabolism in the body. In lieu of any clinical “cure,” the condition is managed through the administration of pharmacological aids, insulin supplements, diet restrictions, exercise, and the like. The conventional clinical prescriptions are limited by their life-long dependency and diminished potency, which in turn hinder the patient’s recovery. This necessitated an alteration in approach and has instigated several investigations into other strategies. As Type 1 diabetes (T1D) is known to be an autoimmune disorder, targeting the immune system in activation and/or suppression has shown promise in reducing beta cell loss and improving insulin levels in response to hyperglycemia. Another strategy currently being explored is the use of nanoparticles in the delivery of immunomodulators, insulin, or engineered vaccines to endogenous immune cells. Nanoparticle-assisted targeting of immune cells holds substantial potential for enhanced patient care within T1D clinical settings. Herein, we summarize the knowledge of etiology, clinical scenarios, and the current state of nanoparticle-based immunotherapeutic approaches for Type 1 diabetes. We also discuss the feasibility of translating this approach to clinical practice.

## 1. Introduction

Diabetes is a chronic health condition that affects the metabolism of glucose in the body due to decreased insulin secretion from the pancreatic islets. Diabetes is further classified into three main types: (i) Type 1, (ii) Type 2, and (iii) gestational diabetes. Out of these, Type 1 diabetes (T1D) is caused by an autoimmune disorder that inhibits the production of insulin in affected individuals [1,2]. Chronic autoimmune diseases are the consequence of the mistaken recognition of self-proteins (antigens) as foreign by the immune system, which leads to an immune response and subsequent destruction of the targeted tissues [3]. T1D is characterized by selective loss of insulin-producing beta cells of islets of Langerhans in the pancreas. Such a loss disrupts the glycemic homeostasis of the body. The loss of beta cell mass occurs when autoreactive T cells migrate to the Langerhans islets and cause local inflammation [4,5]. This autoimmune-mediated beta cell destruction can be asymptomatic for years prior to the manifestation of clinical symptoms. Currently, there is no clinical “cure” of T1D. The condition is managed using insulin and its variates which attempt to maintain the blood glucose levels within the healthy range. The condition furthers needs to be supplemented with proper diet, exercise, and weight management [6,7]. A big limitation of the conventionally prescribed insulin replacement therapy is its life-long dependence on such treatment [8,9,10]. Factors including lack of specificity, altered effects, and diminished potency can also hinder the patient’s recovery.

In recent years, various studies have been undertaken to limit or reverse this autoimmune-mediated beta cell damage and a variety of strategies have been developed to enhance beta cell survival and/or islet regeneration [11,12,13]. Immunotherapy deals with the development of preclinical therapeutic strategies for disease treatment targeted towards the body’s immune system. The immune system comprises a complex collaboration of cells working together to counteract the pathogens which invade the body. This intricacy makes the immunotherapeutic approach a particularly challenging task, as it involves various measures to prevent, diagnose, and treat a variety of disorders [14,15]. These approaches focus on the combination of immunity analysis and clinical chemistry to develop alternative therapeutic methodologies. It has been shown that autoantigen in combination with an appropriate immunomodulator holds potential in tackling a variety of autoimmune diseases [16]. These two compounds, when delivered simultaneously, affect the action of immune cells by modulating their autoreactivity. This immune modulation can play a key role in delaying and reducing the onset of T1D and is being explored using functional nanomaterials [17]. However, the ideal therapeutic approach for T1D should be long-lasting, render elevated antigen-specific tolerance, and integrate personalized or broad antigen specificity.

Towards this end, various nanoparticulate systems have been developed and have been transformative in the field of nanomedicine. Nanotechnology provides us with this tailorable ability to work at the atomic and molecular levels and opportunities to understand and create nanoparticulate platforms with new intrinsic properties attributed to their nanoscale size (<100 nm). For use in biomedical applications, the nanomaterials must exhibit unique properties such as aqueous stability, biocompatibility, and interactive functional groups. A variety of “smart” functional nanomaterials have made significant advances in the areas of drug delivery and drug discovery, biosensing, cell labeling and transplantation, gene therapy, immune therapy, and diagnoses and imaging [18,19,20,21,22,23]. The most attractive trait of these nanosystems is their large surface area/volume ratio, which allows their surface to carry and deliver multiple molecules to the same site of interest. Recent years have seen extensive evaluation and applications of nanosystems such as polymeric, lipid-based, liposomes, dendrimers, and inorganic nanoparticles (Metal oxides, gold, rare earth metals, graphene etc.). The range of nanoparticles used in recent preclinical studies is enormously extensive.

Various materials and compositions are being explored to both target and modulate the specific immune cell populations for the treatment of T1D. However, an absolute “cure” remains a challenge [24]. Insulin, its variates, and other therapeutic molecules which can modulate hyperglycemia have already been extensively delivered using nanoparticles to remediate the condition [25,26,27,28,29]. However, for the use in immunotherapy of T1D, these functional nanomaterials are required to conjugate and deliver immunomodulators to desired localized sites while maintaining its activity. Combing through a wide variety of targets, immunomodulatory agents accumulate locally and initiate specific immune responses. Development of tolerogenic vaccines for T1D and beta cell replacement therapies have shown promise by using nanoparticulate platforms [30,31,32]. By delivering tolerogenic agents and autoantigens, these nanomaterials can also be used to restore and enhance immune tolerance against T1D [33].

**Figure 1:**
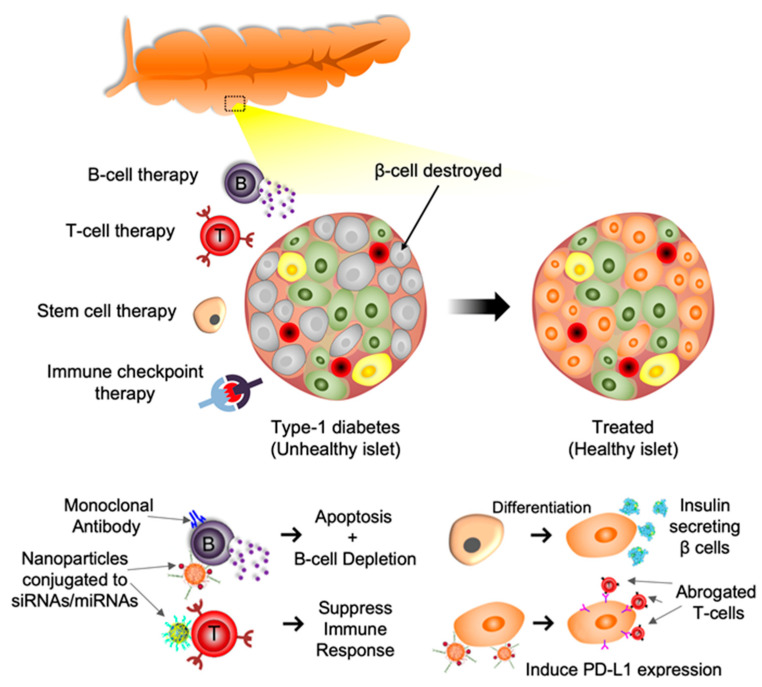
Schematic depiction of immunotherapies for T1D described below. T Cell Therapy: T cells modulated by nanoparticles conjugated to siRNA/miRNA leads to immunosuppression. B Cell Therapy: Modulation of B cells via monoclonal antibodies and/or nanoparticles conjugated to siRNA/miRNA leads to B cell apoptosis and depletion. Immune Checkpoint therapy: Nanoparticles conjugated to siRNA/miRNA induces PD-L1/CTLA4 expression resulting in abrogation of proximal T cells. Stem Cell Therapy: Differentiated stem cells transform into insulin producing beta cells.

In this regard, this manuscript summarizes our knowledge of diabetic etiology, clinical scenarios, and the application of nanoparticle-based delivery approaches in targeting the immune system towards the treatment of T1D. Figure 1 illustrates various immunotherapeutic strategies currently under exploration to combat T1D discussed in the current review.

### 1.1. Understanding T1D: Etiology and Current Clinical Scenario

T1D is a chronic autoimmune disorder characterized by the loss of insulin-producing beta cells within pancreatic islets. The immune-mediated destruction of beta cells results in insufficient levels of insulin production, creating a dependence on exogenous sources of insulin in affected individuals [2,34]. Both genetic and environmental factors contribute to the development of T1D. Typically, genetic predispositions are the result of polymorphic alleles coding for human leukocyte antigen (HLA), insulin gene promoter, and cytotoxic T lymphocyte antigen-4. In-fact these genetic disturbances account for 55–65% of T1D cases [35,36,37]. In the vast majority of T1D cases, pathogenesis is identified by the presence of several pancreatic autoantibodies. These autoantibodies include antibodies to insulin (IAA), islet cell cytoplasmic antibodies (ICA), insulinoma-associated 2 or protein tyrosine phosphatase antibodies (IA-2), zinc transporter8 (ZnT8), and glutamic acid decarboxylase (GAD65). The number of autoantibodies present in circulation and their abundance contribute directly to an individual’s likelihood for developing T1D [38]. Autoantibodies provoke CD4^+^ and CD8^+^ T cells to migrate into pancreatic islets, triggering insulitis. Once these lymphocytes infiltrate the intra-islet space, they proliferate and attack endogenous beta cells, leading to the cessation of insulin production within pancreatic islets and subsequent loss of glycemic homeostatic mechanisms [39,40,41]. Insufficient insulin production prevents the inhibition of lipolysis leading to uncontrolled fat metabolism and the accumulation of ketone bodies in the blood. The buildup of ketone bodies such as acetoacetate and β-hydroxybutyrate causes ketoacidosis. In the absence of compensatory mechanisms, ketoacidosis results in loss of consciousness, cerebral edema, mental confusion, coma, and even death [42].

Studies investigating the molecular mechanisms behind T1D progression have determined that an individual’s predisposition towards developing this autoimmune disorder is controlled by complex interactions between a multitude of genetic loci and environmental factors. As previously mentioned, polymorphic alleles at the HLA locus account for nearly half the familial clustering of T1D. HLA molecules are divided into class I (A, B, and C), and class II (DR, DQ, and DP). While HLA class I molecules are expressed on the surface of nearly all cells, HLA class II molecules are only expressed on the surface of activated T cells, B cells, and antigen-presenting cells [36,43]. Abnormalities affecting the genes which code for class II molecules contribute significantly towards T1D susceptibility, specifically those mapped to HLA-DQ/DR. Haplotypes corresponding to DR3-DQ2 and DR4-DQ8 have been observed in approximately 90% of patients diagnosed with T1D [44,45]. One study revealed that expression of human DR3 and/or DQ8 in non-diabetes-prone mice generated a loss of immune tolerance to the beta cell autoantigen glutamic acid decarboxylase, resulting in spontaneous insulitis [46]. Prior to insulitis, antigen-presenting cells (APCs) carrying beta cell peptides migrate towards lymph nodes located near the pancreas. There they present autoantigens to autoreactive CD4^+^ T lymphocytes. This interaction triggers a signaling cascade leading to the activation of autoreactive CD8^+^ T cells, which in turn infiltrate pancreatic islets. Once inside, CD8^+^ T cells begin lysing beta cells, thereby eliminating the islets’ ability to produce insulin [1,47,48]. Despite this, residual levels of beta cell mass have been detected long after diagnosis, however, the cause of this remains unknown [49].

Prevalence of T1D is dependent on several environmental factors including geographical location [50]. When examining the various components necessary for T1D development, the conjunction between epigenetic forces and genetic predisposition must be properly assessed. Epidemiological studies designed to assess regional T1D pervasiveness have observed elevated prevalence of the autoimmune disease in northern populations. Countries including the U.S., Canada, Finland, and Sweden suffer the highest rates of T1D; this phenomenon is believed to be the result of vitamin D deficiency [51,52,53]. Vitamin D serves an important role in the regulation of immune activity. Metabolites of vitamin D, such as 1,25-dihydroxyvitamin D3 suppress T cell proliferation and alter cytokine expression resulting in a more calculated immune response. Furthermore, vitamin D is known to heighten tolerance towards self-antigens and the development of autoantibodies [54,55]. Such factors should be taken into consideration when developing therapeutics or treatments for T1D. T1D continues to affect a growing population of affected individuals around the world and as a result, more therapies are needed to accommodate this growing complication.

The current clinical scenario for treating T1D is centered around intensive diet treatments and exogenous insulin administration. Clinicians currently utilize a conjunction of insulin pumps and continuous glucose monitors (CGM) to enhance the precision of glycemic control. Despite this, hypoglycemia remains one of the most consequential acute complications associated with insulin replacement therapy. Severe hypoglycemia, often occurring during nighttime hours, can result in seizures, coma, and death. In fact, nearly 6% of T1D related deaths are the result of nocturnal hypoglycemia [56]. Innovations in CGMs and insulin pumps have made significant progress in reducing nocturnal hypoglycemia. The implementation of threshold systems designed to suspend insulin delivery for up to 2 hours until CGM glucose is at a low threshold have displayed promising results in reducing nocturnal hypoglycemia [57]. Although significant progress has been made in this regard, insulin replacement therapy is only so effective at preventing prolonged periods of hyperglycemia and continues to suffer from the risks associated with hypoglycemia. Another roadblock to insulin replacement therapy stems from the complicated processes by which insulin is manufactured and distributed. More recently, insulin analogues such as Lispro, Aspart, and Delgludec insulins have been developed. Such analogues bear higher efficacy, exhibiting less latency and providing a longer duration of action [58,59]. However, in the U.S., the price of insulin and insulin analogues has increased exponentially over the past several decades. Today, monthly out-of-pocket costs for T1D patients can range between $75–2000 depending on insurance coverage and insulin requirements, making proper treatment inaccessible for some individuals [60]. With the life-long insulin replacement therapy required to treat T1D patients, alternative forms of treatment must be developed to address the growing cohort.

T1D is more uncommon than Type 2 diabetes, resulting in a limited understanding of the immunology of T1D when compared to its counterpart. A variety of cell types are involved in the onset of T1D and involve an intricate cascade of interactions between immune cells and the islet beta cells. Immune cells responsible for this autoimmunity participate in both innate and adaptive immune responses. The initial trigger responsible for directing the autoimmune attack is still unclear, however, most researchers attribute it to an individual’s genetic predisposition. Understanding the role genetically directed antigens play is necessary for identifying checkpoints which can be targeted to generate a cure or reversal of T1D phenotype. In T1D, APCs and varying lymphocytes interact to generate immune responses when presenting pathogens [61,62]. These lymphocytes include the more commonly explored T cells, as well as the more unexplored class of B cells.

### 1.2. T Cell Based Therapy

Elaborate studies in the past 10 years have improved our understanding regarding the adverse effects hyperactive T cells have on endogenous beta cells. These potentially pathogenic migratory cells comprise CD4^+^ and CD8^+^ T cell subpopulations, B cells, dendritic cells, and macrophages, which have specificity towards islets of Langerhans. The T cells are held in check by various regulatory mechanisms and by a special T cell population known as regulatory T cells (T_regs_). An imbalance/defect in this control mechanism and/or dysfunctional Treg population might be one of the causes for the onset of T1D [63]. Various attempts have been made in the direction of understanding and identifying the T cell markers which could mediate the strengthening of immunoregulation [64]. Sorting out the molecular profile of the dysfunctional T_reg_ population and introduction of immunomodulatory agents against these markers may reveal promising targets for T cell immunotherapy [65,66,67,68]. The current and developing immunotherapies aim at either preventing the autoimmune response or re-establishing the regulatory control over the autogenic T cell population.

The CD4^+^ T cells do not cause beta cell death through direct contact, but rather secrete cytokines to promote recruitment of other immune cells. These inflammatory cytokines, such as IFNγ, IL-1β, and TNFα, also stimulate beta cell death, thereby aggravating islet loss during T1D. On the other hand, CD8^+^ T cells lead to beta cell death through direct contact with the beta cells [5,69]. CD4^+^ T cells differentiate into a variety of helper T cells, which have their unique cytokine profiles that give them effector functions adapted to a variety of infections [70]. Manipulating these effector or regulatory CD4^+^ T cells response is a promising immunotherapy strategy in various autoimmune disorders. Keeping these factors in mind, Eichmann et al. studied the effects of co-stimulation blockade using abatacept over CD4^+^ memory T cells and the consequent decline in the beta cell function [71]. Their treatment demonstrated a substantial alteration in the population of CD4^+^ cells and T_reg_ cells. Their results also indicated that this approach only affects conventional CD4^+^ but not CD8^+^ T cell populations. Similarly, Long et al. used Teplizumab to enhance the secretion of inhibitory molecules to reduce the population of CD4^+^ and CD8^+^ cells, which delayed the onset of T1D [72]. Autoreactive CD8^+^ T cells have heterogenous phenotypes and their expression is seen to be affected by the rate of progression of T1D [73]. Elevated expression of activated islet-reactive CD8^+^ memory T cells was predominant in T1D patients who demonstrated a rapid loss of C-peptide, while expression of multiple inhibitory markers, limited cytokine levels, and reduced proliferation marked a slower rate of progression of T1D [74].

Identification of markers in correcting the function of dysfunctional T_reg_ cells can also work in the direction of reversal of autoimmune response [75]. These T_reg_-based therapeutic approaches can be helpful to restore tolerance in the T cell-mediated autoimmune responses [66,76]. T_regs_ with phenotype CD8^+^CD25^+^FOXP3^+^ have been seen to effectively suppress the activity of pathogenic T cells and decrease the population of CD8^+^ effector T cells [77]. Serr et al. identified HLA-DQ8-restricted insulin-specific CD4^+^ T cells and demonstrated efficient human insulin-specific Foxp3^+^ T_reg_-induction after sub-immunogenic vaccination with strong agonistic insulin mimetopes in vivo [78]. Functional chimeric antigen receptors (CARs) against insulin in conjunction with FOXP3 can be used to modify naïve effector T cells to specific T_reg_ cells in order to redirect their specificity towards T1D [79]. This approach is expected to result in high specificity, which would minimize the off-target impacts. Modulation and engineering of these T_regs_ also face drawbacks such as insufficient population, stability of modified expression, and antigen specificity.

More recently, nanomedicine has introduced novel techniques which are significantly capable of altering the immune response [80,81,82]. This precise control over the immunomodulation by the use of nanoparticles are proficient in inducing immune tolerance, ranging from triggering the pathogenic T cells to T_reg_ cells, and further into effector T cell populations [83]. One of the approaches where nanoparticles have found their use is by using dextran particles to administer autoantigen and immunosuppressant (rapamycin,) which selectively affect the effector T cells without global immunosuppression [84]. It also resulted in a reduction in the proliferation of CD4^+^ T cells while an increase was observed in the ratio of FOXP3^+^ to IFNγ^+^ T cells. These microparticle-based platforms were effective in altering multiple immune cell functions by selectively inhibiting disease-associated T cell immunity and leaving the general immune responses unbroken. Bergot et al. hypothesized whether the tolerizing immunotherapy with a single peptide might be effective to control T1D, which is guided by multiple antigens [85]. They co-encapsulated an autoantigen (chromogranin A, ChgA) along with 1α,25-dihydroxyvitamin D3 in liposomal bilayer and monitored the specific autoimmune response. Liposome administration subcutaneously, but not intravenously, induced ChgA-specific Foxp3^+^ and Foxp3^−^ PD1^+^ CD73^+^ ICOS^+^ IL-10^+^ peripheral regulatory T cells in prediabetic mice, and liposome administration at the onset of hyperglycemia significantly delayed diabetes progression. Their work deduced that the liposomes encapsulated the single CD4^+^ peptide, and vitamin D3 analogues induce ChgA-specific CD4^+^ T cells that regulate CD4^+^ and CD8^+^ self-antigen specificities and autoimmune diabetes in NOD mice. On similar lines, Jamison et al. fabricated poly(lactide-co-glycolide) (PLG) nanoparticles and loaded with Insulin–ChgA hybrid peptide in order to monitor the balance between effector and regulatory T cells [86]. Administration of hybrid insulin peptide-coupled PLG NPs was found to prevent diabetes by impairing the ability of CD4^+^ T cells to produce proinflammatory cytokines through induction of anergy, leading to an increase in the ratio of Foxp3^+^ regulatory T cells to IFN-γ^+^ effector T cells. It was also observed that interleukin-2 (IL-2) could enhance the T_regs,_ which in turn maintained their control over the pathogenic T cells. Aboelnazar et al. studied this relation as a therapeutic strategy and fabricated IL-2-loaded chitosan nanoparticles [87]. They found that low availability of IL-2 in the cellular microenvironment, an inverse correlation between T_reg_ and natural killer (NK) cell expression which was also related to the expression of FOXP3 on T_reg_ cells. IL-6 receptor-mediated signaling also plays a role in development of T cells, which then take part in T1D pathogenesis. Greenbaum et al. attempted to modulate the T cell phenotypes by blocking IL-6 using tocilizumab [88]. They found that while tocilizumab reduced T cell IL-6 signaling, it did not have any effect on CD4^+^ T cell phenotypes. No significant difference in the slowing of beta cell loss was observed. Antigen-specific T cell immune tolerance can also be induced by the use oof nanoparticles. A conjugated system of carboxylated polystyrene beads (PSB) with an immunomodulating peptide, HLA-A*02:01-restricted epitopes, was seen to successfully induce tolerance and suspend the autoimmune cascade in NOD and transgenic humanized mice [89].

These works are suggestive that engineered nanomaterials can conjugate immunomodulators and target desired precise sites of both adaptive and innate immune responses. The size and surface chemistry of these nanomaterials can be tailored according to the identified target and can be tuned to respond to specific stimuli. Administration of these modified nanoparticles to the T cell family involved in the autoimmune response to T1D can successfully aim to restore immune tolerance and regulatory functions of the immune system. These studies have been tabulated in Table 1 below.

### 1.3. B Cell Based Therapy

It is known that T1D is affiliated with the loss of tolerance by autoreactive (islet-reactive) B cells [90,91]. B lymphocytes (cells), in addition to T lymphocytes (cells), work directly with the adaptive immune system to produce cellular and humoral defense mechanisms for protection against infections or tumors [90,92]. Therefore, the depletion of B cells makes an individual highly vulnerable to opportunistic infections. However, to prevent autoimmunity from occurring, the B cells must be suppressed, rehabilitated, or culled [92]. There is also a correlation between the number of CD20^+^ B cells and a decreasing pancreatic beta-cell count [90]. In the current realm of diabetes treatment, there exists a need to discover effective immunotherapy methods, as current treatment by daily insulin injection(s) is not a sustainable way to treat diabetes and is not a cure. The role of B cells in non-obese mice models has shown to arrest the disease development around the preinsulitis stage [93]. Therefore, B cell immunotherapy is proposed as an effective treatment for T1D patients [61].

To target B cells, monoclonal antibodies are being used to identify surface antigen markers on the surface of the cells. The biomarkers targeted on the B cell surface are those involved in the processes of maturation, differentiation, and survival [61,90,93]. Through mechanisms including, but not limited to, complement-dependent cytotoxicity, antibody-dependent cellular cytotoxicity, and induction of apoptosis, the antibodies are able to induce cell death [61]. Current approaches to antibody B cell therapy include the use of rituximab (RTX), which has proven to show low efficacy in treatment results due to the following factors: detrimental side effects caused by B cell depletion, rapid reemergence of autoreactive B cells post RTX treatment, and the depletion of regulatory cells as collateral of RTX treatment [90]. Pancreas-localized B cells may also be resistant to RTX-mediated deletion, as indicated by a down-regulation of the CD20^+^ surface marker [93]. Due to confounding effects, it is difficult to manipulate the dose and duration of RTX treatment, hindering the effectiveness and clear outcome of the treatment [61]. Combination therapy is another approach used to deplete B cell levels with aims to control glycemia and reverse T1D. Through combination therapy, antigens and antibodies are administered together to achieve a therapeutic outcome that offers better protection than administering the agents alone [62,94,95]. Oral administration of insulin in combination with anti-CD20 antibodies shows low efficacy in reversal when compared to administering proinsulin DNA with an anti-CD20 antibody [94,95]. However, the aforementioned method is moderately effective in the prevention of T1D. Thus, the combination therapy approach to an immunological treatment of diabetes shows (limited) efficacy in the prevention of T1D, and little to no efficacy in the reversal of T1D.

A treatment is needed that can deplete B cells before the onset of hyperglycemia—a point where it is too late to arrest disease progression [62,93]. B cell depletion has been shown to arrest diabetes progression at the pre-insulitis stage, prior to T cell islet infiltration and insulitis development [96]. Further, specificity for autoreactive B cells is needed, so that pan-B cell-depletion is limited, and global immunosuppression does not occur [96,97,98]. The use of a site-specific receptor-mediated drug delivery system, in conjunction with small interfering RNA (siRNA), to target antiapoptotic factor B cell lymphoma/leukemia 2 (BCl2), is an effective method of gene silencing in B cell immunotherapy [99]. This can be achieved through nanomedicine, by using synthetic polymer-based nanoparticles conjugated with siRNA to specifically target autoreactive B cells [100]. Nanomedicine uses nanoscale substances and materials to monitor and treat human biological systems. Nanoparticles (NP’s), a major proponent of nanomedicine, are known to couple various properties with reduced toxicity, in comparison to other bio-interfaces, all at the scale of nanometers [30].

Varying polymers such as chitosan, calcium pectinate zinc oxide, alginate, casein, and other polyester or polycationic acrylic polymers have proven to be effective oral administrative carriers for immunomodulators, insulin, and other engineered vaccines to treat T1D [98]. These polymers are characterized by nanoporous structures, in which various therapeutic agents such as insulin, proinsulin DNA, and siRNAs can be conjugated, and released upon conformational change when glucose levels fluctuate [95,98]. An increase in glucose levels, for example, would induce a conformational change in the nanoparticles, allowing for conjugated materials in the nanopores to be released and directed to the immune cells through a mechanism of biodegradation.

T1D is known to be characterized by the presence of autoantibodies (AABs) and the spreading of islet autoantigens (AAGs) during a prolonged, subclinical period, prior to the detection/diagnosis of T1D, during which it’s expected that seroconversion will occur early in age, with epitope spreading indicative of disease progression [101,102]. The mechanisms leading to the generation of AABs and AAGs, and thus the onset of T1D, are considered genetic, though environmental factors can play a part. Through genetic mechanisms, T cells generate blueprints for the construction of autoreactive B cells, which in turn present beta-cell antigens to autoreactive T cells, creating a vicious loop which aids in the development and progression of T1D [102,103]. Thus, it is essential that an immunotherapeutic treatment for B cell intervention is fabricated to break the detrimental loop which leads to the overall reduction of beta cells, which can be approached by nanomedicine. To induce (therapeutic) genetic expression within the B cell environment, NPs may be enveloped with CRISPR-cas9 (a gene editing system that allows for DNA repair, deletion, or modification) that expresses various cofactors to decrease expression and induce deletion of the autoreactive B cells [103]. In turn, the reduction of autoreactive B cells would result in increased B cell tolerance, and a reduction in the decrease of beta cells. Limited studies have produced results on immunotherapeutic approaches to treating T1D with NPs by targeting B cells, however there are studies that show a successful depletion of B cells in varying autoimmune diseases, leading to the prevention, and, in some cases, a reversal of the disease [101,102,104]. An immunotherapeutic approach to treating T1D through depleting autoreactive B cells poses an effective method for the actual reversal of T1D, which is largely due to individual genetic predispositions. These genetic predispositions may be combated by using CRISPR-cas9 or other immunotherapeutic agents conjugated with the prospective cofactors to induce the cellular and humoral responses, which aims to deplete the autoreactive B cells. It is important to consider the various stages of T1D during immunotherapy, as genetic and environmental conditions will impact the efficacy of the treatment.

Thus, it is indicative that a dynamic nano-delivery system must be constructed that can bind immunotherapeutic agents and release them upon environmental and physiological change. Furthermore, the ability to have controlled release of the agents in the system allows for more tunability in response to varying conditions, environments, and stimuli. Thus, nanoparticles are highly desirable in their role as a vaccine candidate, aiming to break immune tolerance and monitor glycemic activity. In comparison to methods such as surface antigen targeting through monoclonal antibodies, nanoparticle administration to specific sites on autoreactive B cells is gaining more and more attention and relevance in present-day studies, as summarized in Table 2.

### 1.4. Immune Checkpoint Molecules-Based Therapy

Immune checkpoint molecules comprise a group of co-stimulatory and inhibitory proteins used to regulate the body’s immune response within a specific microenvironment. Checkpoints such as CD28, a receptor commonly expressed on the surface of CD4^+^ T cells, binds to one of two molecules (B7.1 and B7.2). This interaction promotes proliferation of CD4^+^ T cells and subsequent migration towards designated target cells [105,106]. Similar processes exist with CD8^+^ T cells, however such stimulatory action relies more heavily on the interaction between molecules such as CD70 and CD137 or CD134 [107]. Contrarily, T cell activation can be suppressed via inhibitory signaling pathways governed by checkpoint proteins including programmed cell death protein 1 (PD-1) and its cognate ligands, programmed cell death ligand 1 (PD-L1) and PD-L2 [108]. When bound to these ligands, PD-1 works to regulate the adaptive immune response by initiating immunosuppressive signals leading to the induction of apoptosis and reduced cell proliferation [109,110,111]. Further examples of co-inhibitory molecules include cytotoxic T lymphocytes-associated antigen 4 (CTLA-4), a CD28 homolog which binds competitively to B7.1/2. Similar to PD-1, CTLA-4 reduces T cell activation via inhibitory signaling pathways [112]. In either case, the relationships between these varied axes serve as mechanisms for acute and precise control of the body’s immune system which poses potential solutions for autoimmune diseases like T1D.

In recent years, immune checkpoint therapy has revolutionized the field of oncology. Many cancer cells possess genetic and epigenetics irregularities allowing them to utilize immune checkpoints to promote survival. Studies have found that a variety of cancer cell types upregulate PD-L1 in response to interferon gamma (IFNγ) as well as other oncogenic signaling pathways [113,114]. Consequently, tumor cells express PD-L1 to abrogate T cell mediated antitumor responses. As a result, therapeutics have been developed to interrupt the PD-1/PD-L1 axis allowing T cells to function more effectively. Such therapeutics utilize monoclonal antibodies (mAb) targeted at immune checkpoints markers (PD-1, PD-L1, and CTLA-4) to inhibit T cell suppression [115,116]. However, immune checkpoint blockades have been linked to spontaneous development of autoimmune diseases. Examples of disease resulting from treatment with anti-PD-L1 antibodies include diabetes mellitus, hepatitis, myasthenia gravis, sarcoidosis, hypothyroidism, endophthalmitis, and various skin rashes [117,118]. This relationship indicates that the absence of co-inhibitory signaling may increase the likelihood that an individual develops one of the aforementioned disorders. Several case studies have been conducted analyzing individuals who have developed late onset T1D in response to immune checkpoint therapy [118,119]. Recognizing this relationship, efforts have been made to assess whether increasing co-inhibitory signaling within pancreatic islets could increase beta cell survival.

Recent studies seeking to better understand T1D progression have determined that PD-L1 is expressed by insulin producing beta cells within pancreatic islets during insulitis. Upregulation of PD-L1 coincides with islet infiltration, as well as other factors such as increased exposure to interferons (IFN) alpha and gamma. Studies analyzing this relationship have determined that the heightened presence of IFNα and IFNγ activates STAT1 and STAT2 transcription factors. This activation corresponds with increased transcription of interferon regulatory factor 1 (IRF1) and subsequent PD-L1 upregulation by pancreatic beta cells [120,121]. Unfortunately, minimal research has been conducted to appraise the potential benefit of increasing PD-L1 or CTLA-4 expression in beta cells to enhance survival during T1D progression. However, preliminary research has demonstrated the protective effect of organ-specific PD-L1 expression in transgenic NOD mice. Wang et al. found that the severity of insulitis in PD-L1 transgenic NOD mice was significantly reduced when compared to controls [122]. Furthermore, islets transplanted into diabetic recipients persisted for a significantly longer period of time when compared to non-transgenic controls. Despite this, development of T1D remained constant between experimental and control groups. Another study attempting to increase survival rates among transplanted human islet-like organoids (HILOs) within NOD mice determined that overexpression of PD-L1 contributed significantly to the HILOs’ survival rate within a diabetic mouse model. Without disturbing insulin production, PD-L1^+^ HILOs maintained glucose homeostasis for more than 50 days whereas PD-L1^-^ HILOs were only able to maintain glucose homeostasis for approximately 10 days [123]. These data present a potential therapeutic benefit to immune checkpoint therapy in T1D.

A significant roadblock when utilizing immune checkpoint proteins in a clinical setting stems from the mechanism by which PD-L1 and CTLA-4 overexpression is induced. One potential solution presents itself in the form of iron-oxide nanoparticles (NPs). Nanoparticles conjugated to various microRNAs (miRNAs) can be used to induce overexpression of co-inhibitory molecules for the purpose of protecting endogenous beta cells [124]. While the literature pertaining to this specific topic is limited, studies attempting to enhance cancer therapeutics have determined that PD-L1 regulation can be achieved via NPs conjugated to miR-200c. Such a combination has proven to inhibit PD-L1 expression, especially when compared to naked miR-200c [125]. Further examples of miRNAs which contribute to the regulation of PD-L1 and CTLA-4 include miR-138-5p, miR-513, miR-200a, and miR-34a [126,127,128]. NPs serve as an ideal delivery vehicle for miRNA-based therapeutic payloads [129]. Contrarily, NPs can be used to deliver antisense oligonucleotides designed to increase expression of co-inhibitory molecules such as PD-L1 and CTLA-4 within pancreatic islets. These immune checkpoint protein molecules currently under investigation have been tabulated together in Table 3 for a better overview.

Despite the potential benefits associated with immune checkpoint therapy in T1D, minimal research has been conducted to further its clinical application. This particular subset of biomolecular research remains dormant while alternative therapeutic avenues are explored. Utilizing the innate mechanisms by which the immune system is stimulated/inhibited could prove useful in the battle against T1D. In conjunction with more traditional forms of treatment, immune checkpoint therapy has the potential to curb the progression of T1D and preserve insulin independence for a more prolonged period of time. Furthermore, such applications may provide clinicians with a more effective form of theranostic-based treatment for T1D patients.

### 1.5. Extracellular Vesicles and miRNA-Based Therapy

Extracellular vesicles (EVs) are membrane-bound small vesicular bodies released by the cells and are utilized in cell-to-cell communication/signaling. These vesicles are relatively small in size and fall under the nanoscale category. However, they preserve the ability to transport molecular cargo. EVs are categorized into three sub-classes, namely, microvesicles, exosomes, and apoptotic bodies. Distinctions are based on their size, type of originating cells, and formation mechanism. They can be released in response to a variety of external stimuli. Examples of this include changes in cell microenvironments (pH, temperature, irradiation), cellular stress, and chemically-induced activation.

EVs may also serve as a communication bridge between immune cells and beta cells. Pancreatic islets have also been shown to secrete EVs that behave in an autocrine manner to regulate beta cell proliferation and death. These islet mesenchymal stem cell-derived exosomes containing miRNAs, can activate the T cell response and stimulate the release of interferon gamma (IFN-γ) to induce autoimmune responses in T1D [130]. Recently, nucleic acids containing exosomes—especially miRNAs—have been shown to regulate communication networks between organs in pathological processes relating to diabetes. One such example includes influencing metabolic signals and insulin signals in target tissues, affecting cell viability, and modulating inflammatory pancreatic cells [131]. This also opens the possibility for exosomes to be developed and utilized as a tool to improve the islet transplant by modulating the immune response or as a biomarker of recurrent autoimmunity for islet transplant diagnosis.

A class of short noncoding RNAs of 19–22 nucleotides, known as microRNAs (miRNAs), act as negative regulators of gene expression by partially pairing to the 3′ or 5′ of the untranslated regions of their target messenger RNAs (mRNAs) [132]. This new and fast rising technology using miRNAs has appealed to many researchers as a potential, minimally invasive biomarker for T1D due to three main reasons: miRNAs are exceptionally stable in cell-free body fluids such as serum, they have high resistance to RNAse digestion, and miRNA molecules have an ability to remain intact in extreme conditions (such as being in extended storage and going through repeated freeze–thaw cycles). Additionally, there is a strong possibility that miRNAs are involved in gene regulation of T1D development [133,134].

**Figure 2:**
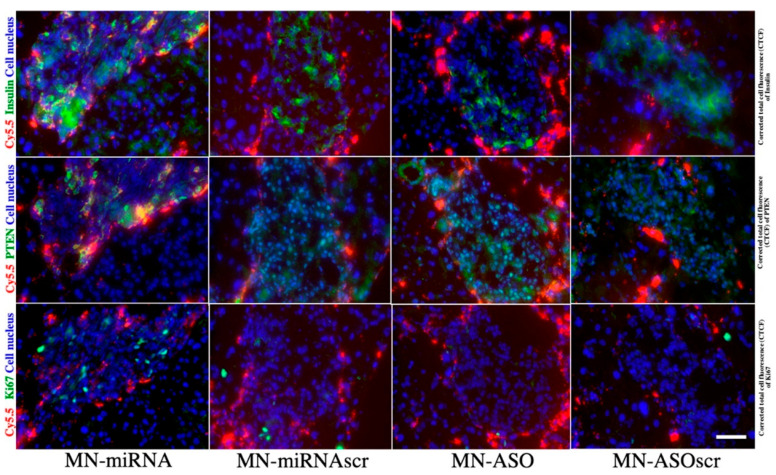
Fluorescence microscopy of consecutive frozen pancreatic sections from STZ-induced diabetic mice injected with MN-miRNA, MN-ASO, MN-miRNAscr, and MN-ASOscr. Animals injected with MN-miRNA showed higher insulin expression in pancreatic islets (top: green, insulin; red, Cy5.5; blue, cell nucleus) when compared to the animals injected with MN-ASO or control nanodrugs. These animals also showed downregulated PTEN expression in their islets (middle: green, PTEN; red, Cy5.5; blue, cell nucleus) when compared to the animals injected with MN-ASO or control nanodrugs. Finally, there was a notably higher cell proliferation in the islets of these animals when compared to controls (bottom green, Ki67; red, Cy5.5; blue, cell nucleus); Magnification bar  =  40 μm. All experiments were performed in triplicates, reproduced with permission from Springer Nature [124].

According to Scherm et al., miRNA expression differs in peripheral mononuclear cells (PMNC) and specific immune cell subsets, such as regulatory T cells, in T1D patients when compared to healthy individuals [135]. This uncharacteristic expression in miRNA leads to disrupted T cell differentiation and loss of function, subsequently resulting in immune activation and the onset of islet autoimmunity and initiation of T1D [136]. In order to provide an elaborate catalog of coding and noncoding miRNAs in human islet-derived exosomes, Krishnan et al. profiled such RNAs in human islet-derived exosomes and identified the RNAs which were aberrantly expressed under cytokine stress [137]. Wang et al. attempted a theranostic approach to deliver miRNA-targeting oligonucleotides conjugated iron oxide nanoparticles in order to modify their expression in pancreatic islets of NOD mice [124]. MiR-216a was identified as a pivotal point in regulating the beta cell proliferation and altering its expression levels significantly affected the progression of T1D (Figure 2). Similarly, modulating the levels of the miR-29 family (miR-29a, miR-29b, and miR-29c) via iron oxide nanoparticles serves to regulate the glucose homeostasis and overcome the hypoglycemic shock induced by diabetes [138]. The levels of miRNA-181a impaired immune tolerance and affect the function of T_reg_ cells. Attempts have been made to successfully block miRNA181a, increasing the T_reg_ induction and reducing the islet autoimmunity in mice [139]. These findings suggest that the identification and subsequent block of trigger markers might allow for the reversal of islet autoimmunity. MiRNAs pertaining to autoantibodies, such as insulin autoantibodies (IAA), islet cell cytoplasmic antibodies (ICA), insulinoma-associated 2, or protein tyrosine phosphatase antibodies (IA-2), zinc transporter8 (ZnT8), and glutamic acid decarboxylase (GAD65), trigger pancreatic T cells to initiate insulitis.

Levels of miRNA in systemic circulation have been proposed as a new class of biomarkers for diagnosis and prognosis of T1D and this has also presented itself as a new target for modulations and therapeutics [140]. There are alterations in serum levels in newly diagnosed T1D patients, with some specific miRNAs appearing to be related to glycemic controls [141]. This newer class of potential circulating biomarkers for T1D have narrowed down their source and improved our knowledge related to the understanding of the molecular functions of these biomarkers. Akerman et al. studied the possible deviations of miRNA levels in the serum of children. They found the serum to be positive for multiple IAAs, and considered these individuals to be at high risk for T1D development [142]. They found that the serum miRNA profiles and autoantibody-positive individuals with high risk of T1D did not differ with respect to healthy, age-matched controls. Some studies have determined that beta cells initiate T1D progression through the activation of various stress pathways. This accelerates the autoimmune-mediated destruction of beta cells and the subsequent loss of insulin-producing mechanisms [130]. The aforementioned study focuses on the need to identify biomarkers in healthy beta cells, which serve as the guiding markers in identifying and monitoring dysfunctional cells. These approaches can not only help to monitor dysfunctional beta cells, but also improve the diagnostics for early detection of T1D. In this context, Bertoccini et al. focused on levels of circulating miR-375, an alleged biomarker of beta-cell death. They observed that an increase in miR-375 was indicative of later onset of T1D, suggesting residual beta-cell function [143]. MiR-375 was directly correlated to the population of viable beta cells that were under autoimmune attack. These results strongly support the potential of miR-375 as an efficient biomarker for T1D diagnosis and prognosis. Bearing this in mind, Lakhter et al. have analyzed the effects of miR-21-5p upregulation on beta cell survival and functionality [144]. Their study determined that the levels of extravesicular-associated miR-21-5p increase significantly in the T1D developing microenvironment and thus, can serve as an efficient biomarker in early T1D detection. However, they noted that utilizing miR-21-5p as an identifying biomarker has limitations due to the abundance of miR-21-5p in circulation, as well as its presence in multiple tissue types. This limits our capability to extrapolate the exact source/reason of the increased levels. Along similar lines, Santos et al. investigated the roles of circulating miR-101-3p and miR-204-5p with respect to T1D progression [134]. Their work concluded that circulating levels of miR-101-3p are higher in T1D patients and healthy individuals with autoantibodies. Based on this data they inferred that miR-101-3p plays an important role in pathways preceding the onset of T1D and can function as an important marker for diagnosis of T1D.

EVs and miRNAs serve as promising biomarker candidates with potential to assist in early T1D diagnosis and prognosis. In comparison to using naked miRNAs, the methods utilizing the conjugated complexes to nanoparticles or nanoscale vesicles have an advantage in terms of ease of administration and in vivo imaging. Although in its nascent stage, this theranostic approach is gaining increased attention and relevance in present-day studies. Table 4 presents variety of miRNA targeting strategies recently studied for T1D.

It has been reported that miRNA expression differs in peripheral blood mononuclear cells and in specific immune cell subsets, such as regulatory T cells, in T1D patients when compared to healthy individuals [135]. This uncharacteristic expression of miRNA leads to disrupted T cell differentiation and loss of function, which subsequently results in immune activation and the onset of islet autoimmunity and initiation of T1D. Researchers have also found that EVs such as islet mesenchymal stem cell-derived exosomes containing miRNAs can activate the T cell response and stimulate the release of interferon gamma (IFN-γ) to induce autoimmune responses in T1D [131].

### 1.6. Stem Cell Targeted Therapy

Stem cell therapy has recently gained further attention and momentum as a promising approach to curing T1D through transplantation of (differentiated) stem cell-derived beta cells that are capable of producing insulin in vivo [145]. This form of regenerative medicine influenced therapy relies on the transplantation of autologous stem cell grafts which can act in immunomodulation or assist in insulin production [146]. The aim of this form of therapy is to provide longitudinal resolve to patients suffering from T1D. This also contributes to the mission of precision medicine by providing a long-term solution to patient complications and removing the reliance on expensive, short term therapies such as insulin injections, which can be difficult to obtain and manage across the stratified sociocultural spectrum in the United States and globally [147]. The value added by clinical translation of stem cell therapy for T1D supersedes previous treatments in the temporal dimension due to its ability to provide functioning beta cells to the patient longitudinally, thus providing for a prolonged period of insulin production during which the patient may not need to rely on other drugs or therapies. Very quickly stem cell therapy can then alter the social landscape of treatment for T1D and similar autoimmune diseases through multiple dimensions by, providing relief to the patient both physiologically and financially. Due to the complexity of autoimmune diseases and their turbulent nature, it is the hope of many scientists that stem cell therapy can provide a long-term resolution to such diseases. It is evident that stem cell therapy has the potential to introduce a great deal of paradigm shifts in the current approach to treatment of autoimmune disease such as T1D.

Currently, there are various approaches to stem cell-based transplantation and therapy for T1D that are being explored in the clinic [148,149,150]. These approaches often involve immunosuppression of the patient to tolerable levels and subsequent transplantation of the autologous stem cells in the patient to avoid immune rejection and further patient autoreactivity [151]. One such study observed the role of autologous nonmyeloablative hematopoietic stem cell transplantation (AHST). Following this treatment, all but 1 of the 15 patients of various gender and age 14 to 31 were able to remain insulin independent for at least 6 months. The study also showed increased C-peptide levels and decreased anti-GAD antibodies, which is a clinically used biomarker for the diagnosis of T1D [148]. This clinical study is evidence of the longitudinal improvement in patient symptoms and physiological complications that result from T1D. Furthermore, it highlights the impact of a combined approach in which immunosuppression followed by AHST is considered the standard. Another study performing autologous stem cell transplantation for the treatment of T1D used mesenchymal stromal stem cells derived from patient umbilical cord, which were transplanted for treatment of T1D [150]. In this study, improvement in C-peptide levels and reduction in insulin dependence was observed for all patients who underwent treatment. This study highlights another type of stem cell, in this case mesenchymal stromal cells, that can be clinically used in transplantation models for diabetic patients. However, it is evident that these models may require imaging tools and modalities that can allow for the visualization of such parameters as transplant density, biodistribution, viability, and immunogenicity [152,153,154]. This can permit both short term and longitudinal monitoring of the transplant in the patient and allow for timely intervention, as is the case in many post-transplant graft loss incidents [155]. It can also allow for the visualization of certain molecular (bio)markers that can indicate the existence of specific cellular states or functions [156,157,158].

Presently, the main modalities used for molecular imaging of stem cells and stem cell transplants are magnetic resonance imaging (MRI) or magnetic particle imaging (MPI) [159,160]. Each of these modalities rely on the utilization of superparamagnetic iron oxide nanoparticles (SPIONs) for targeted molecular imaging of extracellular and intracellular markers. These SPIONs have previously been altered for targeting of immune cells, islet cells, and stem cells amongst many other cell targets [161,162,163]. They have also been explored for monitoring cell transplants longitudinally [164]. This is particularly useful in the context of providing tools for monitoring of stem cell transplants for the treatment of T1D because of the possibility for post-transplant rejection, due to host immune rejection, issues with cell transplant procedure, or cell viability post-transplant in the patient. The use of nanoparticles to monitoring stem cell transplants during treatment of autoimmune diseases such as T1D opens new doors to paradigms of theranostics and precision medicine, as autologous stem cells are typically used for transplantation and therapeutic purposes. The dynamic array of moieties in the domain of radionuclides, small nucleic acids, and antibodies that can conjugate to nanoparticles provide a platform for targeting specific cells from a heterogenous distribution and performing various forms of combined therapy and imaging (theranostics) [165]. In the context of guiding and improving therapeutic outcomes, nanoparticle-based tracking of stem cells has provided a far more effective and reliable method in vivo when compared to conventional methods such as labeling cells with organic dye or directly labeling them with fluorescent probes, because of their optimal magnetic and optical properties. Although there are a wide variety of structural platforms and nanomaterials with which to engineer theranostic nanoparticles such as silicon and quantum dots, the most biocompatible and clinically used tool for contrast enhancement and targeted therapy are SPIONs. Several studies have highlighted the ability of SPIONs to image and track stem cells post-transplantation in both mice and humans. One such study labeled MSCs with SPIONs and encapsulated these in collagen-based microcapsules for monitoring of the cells post-transplantation [166]. Although this study focused on use of SPIONs for monitoring MSC transplantation for the treatment of myocardial infarctions, SPIONs can also be used for the labeling of differentiated beta cells derived from iPSCs for monitoring of transplant thereof in vivo [167]. Wang et al. have shown imaging of endogenous beta-cell mass through targeting of the glucagon like peptide 1 receptor (GLP-1R) [168]. This was done through conjugation of the exendin 4 to magnetic nanoparticles and subsequent injection of this probe in mice. The group was able to show specific accumulation of the probe in GLP-1R expressing endogenous beta cells and indicated the correlation between reduced signal interference with decreasing beta cell mass over time. This approach can be extrapolated to instances of imaging GLP-1R-expressing, stem cell-derived beta cell transplantations. This also provides a mechanism for the direct targeting of endogenous and transplanted beta cells, regardless of origin, to deliver interventional nanodrugs and therapeutic molecules in vivo. Additionally, these SPIONs have enabled the use of an emerging imaging modality of MPI. Prior studies have performed MPI of human islet cells labeled with dextran-coated SPIONs and transplanted under the left kidney capsule of mice [164]. However, the limitations of MPI result mainly from its inability to decipher viable vs. non-viable cell transplants, especially after a brief period of time where dead cells and their nanoparticles can undergo degradation and generate false positive signals that do not originate from live cells [169]. Despite these limitations, nanoparticles are gaining popularity in their use for monitoring such cell transplants and are continuously being explored as a platform.

## 2. Conclusions and Future Perspectives

In the last century, exogenous insulin therapy has transformed diabetes therapeutics in clinical settings. Since T1D has been recognized as an autoimmune disease, efforts have been made to advance our knowledge of disease mechanisms, its progression, and prevention of the associated autoimmune responses. This understanding serves as our base for designing novel therapeutic strategies in the form of targeted immunotherapeutic approaches. This review summarizes a variety of immunotherapy strategies currently being tested and utilized to cure T1D in an effort to improve the quality of clinical treatment provided to the patient. In an intricate cascade of events involving the onset of T1D, various checkpoints have been identified and have shown success in achieving targeted immunotherapy. However, they are still limited in their ability to maintain long-term glycemic homeostasis and normal insulin secretion. Since 70–90% of beta cell mass is dysfunctional or destroyed by the time clinical help is sought, identification of early-stage immunological biomarkers and intervention may be more beneficial in facilitating an early assessment of T1D. Stem cell-based beta cell regeneration approaches also need to be included in such combinatorial treatment methodologies as it pursues the ultimate objective when beta cells have been damaged. Immunotherapies, focused on a beta cell-regenerating agent and an immunomodulator, represent a promising strategy for finding a cure of T1D.

Nanoparticles have already proven their potential in a targeting a variety of disorders as they offer remarkable clinical diagnostic and therapeutic prospects. More recent works have also shown that combining these immunotherapies with nanoparticulate systems possess enhanced functionalities and have the potential to specifically target the immune check points and slow/arrest the rate of T1D progression. The studies show encouraging results in incorporating the nanoplatforms in-line with the existing immunotherapies towards combating T1D. Although in its nascent stage, it is anticipated that this combinational approach would prove to be a promising avenue to achieve a complete reversal and reset of the dysfunctional immune system in individuals with T1D.

## Figures and Tables

**Table 1:** Summary of strategies for targeting and regulating T cell population and function towards T1D.

	APPROACH	TARGET	REFERENCE/S
1.	Teplizumab	CD4^+^ and CD8^+^ cells	[72]
2.	Population alteration	Autoreactive CD8^+^ T cells	[73]
3.	Functional correction	T_reg_ cells	[66,75,76,77]
4.	Chimeric antigen receptors	T_reg_ cells	[79]
5.	Rapamycin	Selective effector T cells and CD4^+^ T cells	[84]
6.	Liposomal formulation of Autoantigen + 1α,25-dihydroxyvitamin D3	ChgA-specific Foxp3^+^ CD4^+^ T cells	[85]
7.	poly(lactide-co-glycolide) nanoparticles loaded Insulin–ChgA hybrid peptide	Balance population of effector and regulatory T cells	[86]
8.	interleukin-2 (IL-2)	T_reg_ cells	[86,87]
9.	tocilizumab	interleukin-2 (IL-6)	[88]
10.	Carboxylated polystyrene beads with peptide HLA-A*02:01-restricted epitopes	Antigen-specific T cell immune tolerance	[89]

**Table 2:** Summary of strategies for targeting and regulating B cell population and function towards T1D.

	APPROACH	TARGET	REFERENCE/S
1.	Rituximab	Autoreactive B cells	[61,90]
2.	Combination therapy (Antigens + Antibodies)	CD20^+^ B cells	[62,94,95]
3.	Nanoparticles + siRNA gene silencing	Autoreactive B cells	[99,100]
4.	Depletion	Autoreactive B cells	[101,102,104]
5.	Nanoparticles + CRISPR-cas9 (Gene editing)	Autoreactive B cells	[103]

**Table 3:** Summary of strategies for identifying and targeting immune checkpoint markers towards T1D.

	APPROACH	TARGET	REFERENCE/S
1.	CD8^+^ T cell activation	CD70 and CD137 or CD134	[107]
2.	T cell suppression	programmed cell death protein 1 (PD-1) + ligand (PD-L1, PD-L2) upregulation	[108,109,110,111]
3.	T cell suppression	cytotoxic T lymphocytes-associated antigen 4 (CTLA-4) upregulation	[112]
4.	PD-L1 upregulation	Interferons: IFNα and IFNγ	[120,121]
5.	transplanted human islet-like organoids (HILOs)	PD-L1 upregulation	[123]
6.	SPIONs + miRNA	overexpression of co-inhibitory molecules	[124]
7.	Nanoparticles + miRNA (miR-200c, miR-138-5p, miR-513, miR-200a, and miR-34a)	PD-L1 and CTLA-4 regulation	[126,127,128]

**Table 4:** Summary of strategies for targeting microRNAs towards T1D.

	Approach	Target	Reference/S
1.	SPIONs + miR-216a	Expression modulation	[124]
2.	SPIONs + miR-29 family	miR-29a, miR-29b, and miR-29c levels’ modulation	[138]
3.	T_reg_ induction	Block miRNA181a	[139]
4.	Diagnosis and prognosis of T1D	miRNA in systemic circulation	[140]
5.	Biomarker of beta-cell death	Circulating miR-375	[143]
6.	Beta cell survival	miR-21-5p upregulation	[144]
7.	Diagnosis of T1D progression	circulating miR-101-3p and miR-204-5p	[134]

## Data Availability

Not applicable.
